# Association analysis of polymorphisms of the CRHR1 gene with infantile spasms

**DOI:** 10.3892/mmr.2015.3751

**Published:** 2015-05-07

**Authors:** GUANG YANG, LI-PING ZOU, JING WANG, XIU-YU SHI, XIAO-FAN YANG, BIN WANG, YU-JIE LIU, YAN-HONG SUN, FEI-YONG JIA

**Affiliations:** 1 Department of Pediatrics, Chinese PLA General Hospital, Beijing 100853, P.R. China; 2 Department of Neurology, Beijing Children’s Hospital, The Capital Medical University, Beijing 100045, P.R. China; 3 Department of Pediatrics, Cangzhou Central Hospital, Cangzhou, Hebei 061001, P.R. China; 4 Department of Pediatrics, First Hospital of Jilin University, Changchun, Jilin 130021, P.R. China

**Keywords:** infantile spasms, CRHR1 gene, single nucleotide polymorphism, haplotype

## Abstract

While >200 types of etiologies have been shown to be involved in the pathogenesis of infantile spasms, the pathophysiology of infantile spasms remains largely elusive. Pre-natal stress and hypothalamic-pituitary-adrenal axis dysfunction were shown to be involved in the development of infantile spasms. To test the genetic association between the CRHR1 gene, which encodes the corticotrophin-releasing hormone (CRH) receptor, and infantile spasms, five single nucleotide polymorphisms (SNPs) in the CRHR1 gene were genotyped in a sample set of 128 cases with infantile spasms and 131 healthy controls. Correlation analysis was performed on the genotyped data. Under the assumption of the dominant model, the selected five SNPs, rs4458044, rs171440, rs17689966, rs28364026 and rs242948, showed no association with the risk of infantile spasms and the effectiveness of adrenocorticotropic hormone treatment. In addition, subsequent haplotype analysis suggested none of them was associated with infantile spasms. In conclusion, the experimental results of the present study suggested no association between the CRHR1 gene and infantile spasms in a Chinese population.

## Introduction

Infantile spasms, also known as the West syndrome, constitute a unique, age-specific epileptic encephalopathy of early infancy and are characterized by epileptic spasms with psychomotor regression and electroencephalographic (EEG) indication of hypsarrhythmia (1,2). The incidence of infantile spasms has been estimated to be ~0.31 per 1,000 live births (3). The peak age of onset of infantile spasms is three to seven months, and the condition is slightly more common in males (4). A number of underlying disorders, including perinatal brain injury, metabolic disorders and chromosome anomaly, may be the etiologies of infantile spasms (5). However, in ~20% of the cases, no causes are identified (6).

While >200 types of etiologies have been involved in the pathogenesis of infantile spasms, the pathophysiology of infantile spasms remains largely elusive (7). Previous studies showed that pre-natal adverse stress is associated with infantile spasms in animal models and clinical epidemiological analyses (8,9). Shang *et al* (8) found that the onset risk of infantile spasms correspondingly increased with the degree of maternal pre-natal stress. The stress may act on the developing brain and results in the dyssecretosis of the hypothalamic-pituitary-adrenal (HPA) axis and high levels of the corticotrophin-releasing hormone (CRH), thereby causing spasms (10). Baram and Schultz (11) established a CRH rat model to verify the HPA dysfunction hypothesis. CRH administered into the cerebral ventricles of rats during the first post-natal week caused a specific and stereotyped sequence of behaviors: Rhythmic chewing and licking were followed by ‘limbic’-type seizures. This condition suggested that CRH may be a strong inducer of spasms, and its excessive release may be the final common pathway through which infantile spasms were induced by various factors. On the other hand, 50–80% of infantile spasm cases can be successfully treated with adrenocorticotropic hormone (ACTH), although infantile spasms are difficult to control with most conventional anti-epileptic drugs (12). The clinical response of infantile spasms to ACTH can be explained by the suppression of CRH production and release via a negative feedback mechanism (13).

The effect of CRH is mediated via CRH receptors in the cell membrane of effector organs (14). Two major CRH receptors, CRHR1 and CRHR2, have been identified and have functional differences between them (15). CRHR1 is a G protein-coupled receptor localized in frontal cortical areas, forebrain, brainstem, amygdala, cerebellum and the anterior pituitary (16). CRHR1 has a key role in the regulation of the HPA axis in response to stressful events, mediating the action of CRH on the pituitary gland to release ACTH that stimulates the production of cortisol in the adrenal cortex (17). Previous studies have shown that polymorphisms in the coding and regulatory regions of CRHR1 gene are associated with the onset of certain neuropsychiatric disorders, including major depression, bipolar disorder, alcoholism and others (18–20). However, whether genetic variants of CRHR1 are associated with infantile spasms, has not been studied in depth. In the present study, the CRHR1 gene was analyzed as a candidate to test its association with the onset of infantile spasms, as well as its responsiveness to ACTH treatment.

## Materials and methods

### Study subjects

A total of 128 patients with infantile spasms and 131 healthy controls were recruited to perform the case-control analysis. The cases were unrelated Chinese individuals (83 boys, 45 girls; mean age, 6.8±2.9 months), recruited between January 2006 and May 2010 from the Chinese PLA General Hospital and Beijing Children’s Hospital affiliated to Capital Medical University (Beijing, China). The diagnostic criteria for infantile spasms were as follows: 1) The presence of epileptic spasms; 2) hypsarrhythmia or modified hypsarrhythmia indicated by EEG prior to treatment; and 3) onset of the infantile spasms at an age between one month and two years (12). According to etiologies, infantile spasms were classified as symptomatic or cryptogenic, as defined by the International League Against Epilepsy (21). Clinical evaluation was performed by experienced pediatric neurologists. Individuals were excluded from the study if they had received hormone therapy within 28 days prior to recruitment or if they displayed contraindications to hormone therapy. The control subjects were all unrelated Chinese individuals (87 boys, 44 girls; mean age, 7.5±3.7 months) without any nervous system diseases, particularly epilepsy, or family history of epilepsy. No statistical difference existed between the patients and controls in terms of gender and age (Table I). All subjects of the present study were Han Chinese, and their parents were asked to sign written informed consent prior to enrolement in this study. The study was approved by the Committee on Human Study of Beijing Children’s Hospital affiliated to Capital Medical University (Beijing, China).

### Evaluation of effectiveness of ACTH treatment

Patients with infantile spasms received ACTH (Shanghai First Biochemical Pharmaceutical Co., Shanghai, China) treatment 25 U/day intravenously for 3 weeks. ACTH response was evaluated mainly based on changes in seizure frequency. Baseline seizure frequency was assessed by parents or carers. Three weeks after ACTH treatment initiation, the seizure frequency was assessed again. Changes in seizure frequency were classified as seizure-free (cessation of seizure for ≥7 days), reduced (reduction of the baseline frequency by 50-<100%), unchanged (reduction of the baseline frequency by <50%), or increased (>100%). The rate of response, defined as a cessation and reduction of the baseline seizure frequency by ≥50%, was calculated in each group. No change or increased frequency was defined as no response.

### Selection of single nucleotide polymorphisms (SNPs)

The human CRHR1 gene (NM_004382) contains 13 exons and spans 51.5 kb of the sequence on chromosome 17. Based on the HapMap Genome Browser (Phase 1 and 2 – full dataset; http://hapmap.ncbi.nlm.nih.gov/), the tagSNP and functional SNP strategy with a minor allele frequency (MAF)>0.1 were used on a Chinese population, and five SNPs were selected for genotyping in the present study. One SNP, rs28364026, in the 3′ untranslated region (3′UTR), one SNP, rs242948, in the 3′ neargene region, and all other three SNPs were in introns.

### Genotyping assay

Genomic DNA was extracted from peripheral blood lymphocytes using the Qiagen QIAamp DNA Blood mini kit (Qiagen Inc., Hilden, Germany) according to the manufacturer’s instructions. Genotyping primers were designed using the Primer3 software (http://frodo.wi.mit.edu/cgi-bin/primer3/primer3_results.cgi), and the sequences are listed in Table II. Multiplex polymerase chain reactions (PCR) were used to amplify all fragments in a total reaction volume of 20 *μ*l. The amplification reactions contained 1X HotStarTaq buffer, 3.0 mM Mg^2+^, 0.3 mM desoxynucleoside triphosphate, 1U HotStarTaq polymerase (Qiagen Inc.), 10 ng sample DNA and 1 *μ*l multiple PCR primer. All primers were supplied by Beijing SaiBaiSheng Gene Technology Co., Ltd. (Beijing, China). The PCR amplification was performed using a PTC-200 thermocycler (MJ Research, Watertown, MA, USA). The cycling conditions for PCR in all reactions included an initial activation step at 95°C for 15 minutes, followed by 11 cycles of denaturation at 94°C for 20 seconds, touchdown annealing at 65°C for 40 seconds −0.5°C per cycle, extension at 72°C for 90 seconds, followed by another 24 cycles of denaturation at 94°C for 20 seconds, annealing at 59°C for 30 seconds, extension at 72°C for 90 seconds, and finally, extension at 72°C for 4 minutes. After the completion of PCR amplification, phosphatase alkaline shrimp (SAP) and exonuclease I (Exo I) were used to purify the PCR products. One unit of SAP and 1 unit of Exo I were added to 5 *μ*l PCR mixture. The mixture was incubated at 37°C for 1 hour, followed by inactivation at 75°C for 15 minutes. Snap-shot multiplex single-base extension was performed using the SnaPshot Multiplex kit (Applied Biosystems, Life Technologies, Thermo Fisher Scientific, Waltham, MA, USA) with five different extension primers. The 10-*μ*l extension mix contained 5 *μ*l SnaPshot Multiplex reagent, 2 *μ*l purified multiplex PCR products, 1 *μ*l extension primer mix and 2 *μ*l double distilled H_2_O. The extension PCR cycling conditions included an initial activation step at 96°C for 1 minute, followed by 28 cycles of denaturation at 96°C for 10 seconds, annealing at 50°C for 5 seconds and extension at 60°C for 30 seconds. The 10-*μ*l extension PCR product obtained by extension PCR was purified with 1U SAP at 37°C for 1 hour, followed by an inactivation at 75°C for 15 minutes. Pyrosequencing was performed on the ABI3130XL sequencer (Applied Biosystems). Data were collected and analyzed using GeneMapper 4.0 (Applied Biosystems).

### Statistical analysis

Differences in gender as well as frequencies of alleles and genotypes between cases and controls were tested using Pearson’s χ^2^ test. An independent-samples T test was used to examine the difference in age between controls and cases. Pearson’s χ^2^ test was also employed to examine the different allelic frequencies of ACTH effectiveness and etiology in cases. The Hardy-Weinberg equilibrium test was performed for every SNP in the controls. The linkage disequilibrium (LD) blocks were reconstructed with D′ and r2 using Haploview software (http://www.broadinstitute.org/haploview) and pairwise linkage disequilibrium values D’ and r2 were calculated in the control population using the maximum likelihood method. Haplotype blocks were defined using the method of Gabriel *et al* (22). Each samples’ haplotype and dihaplotype was estimated from the genotype data using PHASE 2.0.2 software (available on request). Odds ratios (OR) and 95% confidence intervals (95% CI) were calculated using unconditional logistic regression analysis to evaluate the association between the risk of infantile spasms and each SNP or haplotype or dihaplotype, and adjusted to age and gender. All statistical tests were performed using SPSS 20.0 software (International Business Machines, Armonk, NY, USA). Two-sided P-values<0.05 were considered to indicate statistically significant differences between values.

## Results

In the present study, 128 cases with infantile spasms and 131 controls were examined who were all unrelated Han Chinese children. There was no significant difference between cases and control group regarding gender and age (P>0.05). Table I shows basic data on the subjects.

The positions and MAFs of five SNPs of the CRHR1 gene in the National Center for Biotechnology Information database for Han Chinese in Beijing are shown in Table III. Each SNP was examined with Hardy-Weinberg equilibrium among controls, and no significant difference was observed (P>0.05). Hence, the population was qualified for further analyses. The genotyping rates of five SNPs were all >99.6%.

**Figure 1:**
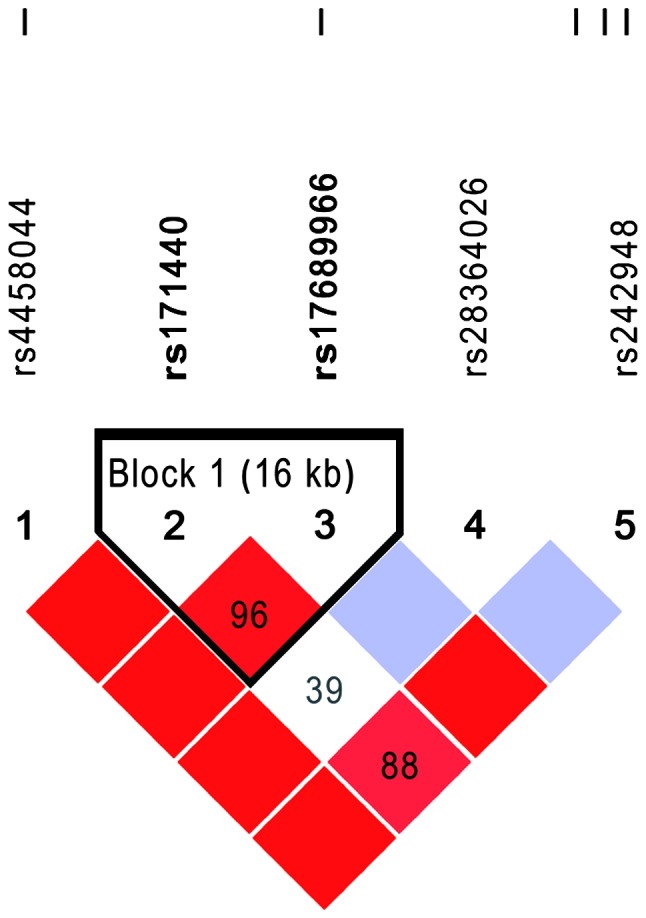
Reconstructed linkage disequilibrium map using five single nucleotide polymorphisms of CRHR1. The haplotype block was determined using haploview software. rs numbers are stated on top, and the numbers in the squares represent the D’ values.

The association of five SNPs in the CRHR1 gene was examined in a dominant model adjusted to age and gender, but no significant differences were identified by Pearson’s χ^2^ test between cases and controls regarding each SNP (Table IV). The LD block of the five SNPs was reconstructed using the genotyping data of the controls with the haploview software. Fig. 1 shows the LD blocks among five SNPs, where two SNPs, rs171440 and rs17689966, were able to form an LD block. PHASE software was used to estimate the possible haplotypes and diplotypes between rs171440 and rs17689966. There were three haplotypes and three common diplotypes, but none of them was found to be associated with the risk of infantile spasms (Table V).

A total of 97 patients with infantile spasms received ACTH treatment for three weeks, among which 62 cases (63.9%) showed responses and 35 cases (36.1%) were unresponsive. The association of ACTH responsiveness and the genotypes of SNPs was analyzed. Under the assumption of a co-dominant model, there was no association of the effectiveness of ACTH and the etiology of infantile spasms with any single SNP (Table VI).

## Discussion

Infantile spasms are a devastating epileptic syndrome in children and have unique features, including characteristic epileptic spasms during infancy, specific electrographic hypsarrhythmia and the arrest of psychomotor development (23). However, the exact pathophysiology of infantile spasms has remained elusive. In the CRH-excess hypothesis, an excessively abnormal secretion of CRH induced by different stressors in the developing brain is thought to increase excitatory amino acid neurotransmission, thereby causing spasms (24). ACTH being the only effective drug for infantile spasms apart from vigabatrin indicates that infantile spasms may be fundamentally different from other epileptic syndromes (1). The clinical response to ACTH can be explained by the suppression of CRH production through negative feedback (10). Based on the effectiveness of ACTH treatment and the HPA axis dysfunction hypothesis, Shi *et al* (25) have proposed a pre-natal stress exposure hypothesis, stating that diverse etiological factors are the onset foundations of infantile spasms, whereas adverse stress during the perinatal period is the onset condition of infantile spasms.

In spite of the large amount of available studies on infantile spasms, genetic studies on the association between polymorphisms of genes and infantile spasms are limited. SNPs are the most common genetic variations with a frequency >1% in humans. Liu *et al* (26) found that the haplotype TCCT in the MC2R promoter is strongly associated with the responsiveness of ACTH therapy in patients with infantile spasms. Further, experiments were conducted to evaluate the function of TCCT *in vitro*, demonstrating that TCCT leads to an increased expression of MC2R and a strong response to ACTH (27). Ding *et al* (28) also reported that the response to ACTH treatment in the CTA haplotype of GRIN1 in homozygous-carriers was higher than that in heterozygous-carriers and non-carriers.

The CRHR1 gene, located at 17q12-q22, encodes a G protein-coupled receptor that binds neuropeptides of the CRH family, which are major regulators of the HPA pathway (29). The encoded protein is essential for the activation of signal transduction pathways that regulate diverse physiological processes, including stress, reproduction, immune response and obesity (30). Polymorphisms in the CRHR1 gene have been implicated in the susceptibility for certain neuropsychiatric disorders, particularly under conditions of stress, including major depression, alcoholism and child abuse (31–33). Geng *et al* (31) found that a functional polymorphism in the 3′UTR of the CRHR1 gene (rs28364032) and three haplotypes containing it showed significant associations with anti-depressant remission. Haplotype analyses of the CRHR1 gene in 2,533 unrelated Caucasian individuals identified one haplotype in the proximal block 1 and two haplotypes in the distal block 2 that showed nominally significant genotype – traumatic stress interactive effects on the likelihood of developing alcoholism (corrected P<0.025) (32). Grabe *et al* (33) reported an association between childhood abuse and the TAT-haplotype of the CRHR1 gene and adult depression, therefore connecting childhood adversities and genetic susceptibility to neurological disorders. The present study hypothesized that CRHR1 is involved in the development of infantile spasms, and to the best of our knowledge, no studies have been conducted regarding the association between the polymorphisms of CRHR1 and infantile spasms to date.

In the present study, CRHR1 was selected as a candidate gene to investigate its association with the risk of infantile spasms and the effectiveness of ACTH treatment. Although cryptogenic cases may rather be associated with hereditary susceptibility, symptomatic and cryptogenic cases were all enrolled in the present study, as not all symptomatic pathogens, including perinatal asphyxia, congenital malformations and tuberous sclerosis, will result in infantile spasms. Thus, genetic factors may also contribute to symptomatic disorders, which has indeed been confirmed by previous studies (34). Under the assumption of the dominant model, the selected five SNPs, rs4458044, rs171440, rs17689966, rs28364026 and rs242948, showed no association with the risk of infantile spasms and the efficacy of ACTH treatment. In addition, subsequent haplotype analysis suggested that none of them was associated with the risk of infantile spasms.

In spite of the strong rationale of the present study, the case-control results did not reveal any statistically significant associations between polymorphisms in the CRHR1 gene and infantile spasms. It is, however, possible that rare variants in the CRHR1 gene may have a role in the onset of infantile spasms and the effect of such polymorphisms may not have been detected in the relatively small population of infantile spasm cases enrolled in the present study. In order to detect the effect of rare variations in the CRHR1 gene using a case control methodology, a significantly larger number of infantile spasms and control individuals would be required to be analyzed. Alternatively, it may be possible to identify rare but important variations in the CRHR1 gene by directly sequencing regions of the CRHR1 gene in a smaller sample of infantile spasms and control individuals.

In conclusion, the present study presented a preliminary evaluation of the role of variations in the CRHR1 gene in infantile spasms. It was the first attempt to study the impact of polymorphisms in the CRHR1 gene in Chinese individuals with infantile spasms. While the present study failed to detect any significant associations between individual SNPs or haplotypes in the CRHR1 gene with the infantile spasm phenotype, it is still possible that variations in this gene may impact the development or progression of this illness.

## Figures and Tables

**Table I:** Distribution of selected variables in cases (n=128) and controls (n=131).

Variable	Cases	Controls	P-value
Gender			0.79^a^
Male, n (%)	83 (64.8)	87 (66.4)	
Female, n (%)	45 (35.2)	44 (33.6)	
Age (months)	6.8±2.9	7.5±3.7	0.88^b^
ACTH responsiveness			
Response (n)	62	63.9	
No response (n)	35	36.1	
Etiology			
Symptomatic (n)	79	70.5	
Cryptogenic (n)	33	29.5	

Age is expressed as the mean ± standard deviation.

a Two-sided χ^2^ test;

b independent-samples T test. ACTH, adrenocorticotropic hormone.

**Table II:** Primer information of NR3C1 for snapshot assay.

rs number	Direction	Primer sequence	Tm (°C)	Product size (bp)
rs171440	Forward Reverse	GGATGGGTCTGTTCCAGGGTGT TGGTCCCCTGCTCTGTAGCCTAA	66.09 65.96	214
rs4458044	Forward Reverse	TGGGCTCCCCTCTTCTGTGAG CTGCCTTCCCTTCCCTCCTCTT	65.66 64.42	106
rs17689966	Forward Reverse	CCTTCTGCCAGGGTTGGAATTG ATCATGGGGCCCTGGTAGATGT	64.63 64.92	212
rs28364026	Forward Reverse	CTGCCTGTGGAGGTGACCTGTT CCCCATTTCAATTCATTCCCATGT	63.68 64.11	158
rs242948	Forward Reverse	ATGGGTCAGGGGAAGGAACAAA TGGAGACAGCTGCATTCACAGC	64.59 64.70	176

**Table III:** Information on five genotyped SNPs of CRHR1 (OMIM^a^ no. 122561; locus 17ql2-q22).

NCBI SNP ID	Chromosome position^b^	Location in gene region	Base change	MAF			P-value^d^	P-value for HWE^e^	Genotyping rate (%)
				Database^c^	Cases	Controls			
rs4458044	43873727	intron	G>C	0.15	0.305	0.294	0.79	0.58	100
rs171440	43893487	intron	C>T	0.267	0.125	0.126	0.97	1	100
rs17689966	43910455	intron	G>A	0.238	0.172	0.181	0.79	1	99.6
rs28364026	43912294	3′UTR	G>A	0.261^f^	0.207	0.202	0.89	1	100
rs242948	43913544	3′neargene	C>A	0.227^f^	0.172	0.164	0.81	0.94	100

a http://www.ncbi.nlm.nih.gov/Omim;

b SNP position according to NCBI dbSNP databases;

c from dbSNP databases;

d P-value for different distributions of alleles between cases and controls;

e P<0.05, compared with the controls;

f The frequency is Chinese Han in Beijing + Japanese in Tokyo. dbSNP, single nucleotide polymorphism database; OMIM, online Mendelian inheritance in man; MAF, minor allele frequency; NCBI, National Center for Biotechnology Information; HWE, Hardy-Weinberg equilibrium; UTR, untranslated region.

**Table IV:** Genotypes of five single nucleotide polymorphisms of CRHR1 and their associations with infantile spasms.

Genetic model	Genotype	Controls, n (%)	Cases, n (%)	P-value^a^	Logistic regression^b^	
					OR (95% CI)^c^	P-value^d^
rs4458044						
Co-dominant	GG GC CC	67 (51.1) 51 (38.9) 13 (10)	57 (44.5) 64 (50) 7 (5.5)	0.13	1.00 1.47 (0.88–2.45) 0.63 (0.23–1.68)	0.14 0.35
Dominant	GC+CC	64 (48.9)	71 (55.5)	0.29	1.30 (0.80–2.12)	0.29
rs171440						
Co-dominant	AA AG GG	100 (76.3) 29 (22.1) 2 (1.6)	96 (75) 32 (25) 0	0.33	1.00 1.15 (0.65–2.05)	0.64 0.99
Dominant	AG+GG	31 (23.7)	32 (25)	0.80	1.08 (0.61–1.91)	0.80
rs17689966						
Co-dominant	AA AG GG	87 (66.9) 39 (30) 4 (3.1)	88 (68.8) 36 (28.1) 4 (3.1)	0.78	1.00 0.92 (0.53–1.60) 0.98 (0.24–4.06)	0.77 0.97
Dominant	AG+GG	43 (33.1)	40 (31.2)	0.75	0.93 (0.55–1.57)	0.78
rs28364026						
Co-dominant	GG GA AA	83 (63.4) 43 (32.8) 5 (3.8)	79 (61.7) 45 (35.2) 4 (3.1)	0.90	1.00 1.10 (0.66–1.86) 0.85 (0.22–3.29)	0.71 0.81
Dominant	GA+AA	48 (36.6)	49 (38.3)	0.79	1.08 (0.65–1.78)	0.77
rs242948						
Co-dominant	TT TG GG	92 (70.2) 35 (26.7) 4 (3.1)	88 (68.8) 36 (28.1) 4 (3.1)	0.97	1.00 1.09 (0.63–1.90) 1.03 (0.25–4.26)	0.76 0.97
Dominant	TG+GG	39 (29.8)	40 (31.2)	0.80	1.08 (0.63–1.85)	0.77

a P-value for genotype frequencies in cases and controls using the two-sided χ^2^ test;

b Adjusted by age and gender.

**Table V:** Association of haplotypes and diplotypes with risk of infantile spasms.

Types	Controls, n (%)	Cases, n (%)	Logistic regression^a^	
			OR (95% CI)	P-value^b^
Haplotypes^c^				
AA	214 (82)	212 (82.8)	1.00	
AG	15 (5.7)	12 (4.7)	0.82 (0.37–1.80)	0.62
GG	32 (12.3)	32 (12.5)	1.01 (0.60–1.71)	0.98
Diplotypes^c^				
AA/AA	88 (67.2)	88 (68.8)	1.00	
AA/AG	12 (9.2)	8 (6.3)	0.68 (0.26–1.78)	0.43
AA/GG	26 (19.8)	28 (21.9)	1.08 (0.59–2.00)	0.80
Others^d^	5 (3.8)	4 (3)	0.80 (0.21–3.08)	0.74

a Adjusted by age and gender;

b P-value from unconditional logistic regression analysis;

c haplotypes and diplotypes were composed of two single nucleotide polymorphisms, rs171440 and rs17689966;

d others: Diplotypes, AG/GG and GG/GG, with frequencies <0.03. OR, odds ratio; CI confidence interval.

**Table VI:** Association of the effectiveness of ACTH and etiology of infantile spasms with single nucleotide polymorphisms of CRHR1.

Genetic model	Genotype	ACTH		P-value^a^	Logistic regression^b^ OR (95% CI)	P-value^c^	Etiology		P-value^a^	Logistic regression^b^ OR(95% CI)	P-value^c^
		Effective n(%)	Ineffective n(%)				Symptomatic n(%)	Cryptogenic n(%)			
rs4458044											
Co-dominant	GG GC CC	27 (43.5) 32(51.6) 3 (4.9)	16 (45.7) 18 (51.4) 1 (2.9)	0.89	1.00 0.97 (0.42–2.28) 0.53 (0.05–5.64)	0.95 0.60	37 (46.8) 38(48.1) 4(5.1)	15 (45.5) 17(51.5) 1 (3.0)	0.87	1.00 1.14(049–2.62) 0.65 (0.07–647)	0.76 0.71
Dominant	GC+CC	35 (56.5)	19 (54.3)	0.84	0.93(0.40–2.16)	0.87	42(53.1)	18 (54.5)	0.90	1.09(048–248)	0.84
rs171440											
Co-dominant	AA AG	14 (22.6) 48 (77.4)	9 (25.7) 26 (74.3)	0.73	1.00 1.16(0.44–3.05)	0.77	58 (73.4) 21 (26.6)	26 (78.8) 7(21.2)	0.55	1.00 0.69(0.26–1.84)	0.46
rs17689966											
Co-dominant	AA AG GG	45 (72.6) 14 (22.6) 3 (4.8)	23 (65.7) 12 (34.3) 0	0.23	1.00 1.66(0.65–423) 0	0.29 0.99	53(67.1) 24 (30.4) 2 (2.5)	24 (72.7) 7(21.2) 2(6.1)	0.44	1.00 0.56(0.21–1.53) 2.19(0.28–17.13)	0.26 0.46
Dominant	AG+GG	17 (27.4)	12 (34.3)	0.48	1.35(0.55–3.32)	0.59	26 (32.9)	9 (27.3)	0.57	0.68(0.27–1.72)	0.42
rs28364026											
Co-dominant	GG GA AA	41 (66.1) 19 (30.6) 2 (3.3)	19 (54.3) 14 (40) 2 (5.7)	0.49	1.00 1.58(0.65–3.83) 242(0.31–18.94)	0.31 0.40	48 (60.8) 29 (36.7) 2 (2.5)	21 (63.6) 10 (30.3) 2(6.1)	0.57	1.00 0.76(0.31–1.85) 2.69 (0.35–20.97)	0.54 0.35
Dominant	GA+AA	21 (33.9)	16 (45.7)	0.25	1.66(0.79–3.89)	0.25	31 (39.2)	12 (36.9)	0.78	0.87 (0.37–2.03)	0.74
rs242948											
Co-dominant	TT TG GG	45 (72.6) 14 (22.6) 3 (4.8)	23 (65.7) 12 (34.3) 0	0.23	1.00 1.66(0.65–423) 0	0.29 0.99	53(67.1) 24 (30.4) 2 (2.5)	24 (72.7) 7(21.2) 2(6.1)	0.44	1.00 0.56(0.21–1.53) 2.19(0.28–17.13)	0.26 0.46
Dominant	TG+GG	17 (27.4)	12 (34.3)	0.48	1.35(0.55–3.32)	0.52	26 (32.9)	9 (27.3)	0.56	0.68(0.27–1.72)	0.42

a P-value for genotype frequencies in cases and controls using two-sided χ^2^ test;

b adjusted by age and gender;

c P-value from unconditional logistic regression analysis; OR, odds ratio; CI confidence interval; ACTH, adrenocorticotropic hormone.
